# Accuracy of non-invasive prenatal testing using cell-free DNA for detection of Down, Edwards and Patau syndromes: a systematic review and meta-analysis

**DOI:** 10.1136/bmjopen-2015-010002

**Published:** 2016-01-18

**Authors:** Sian Taylor-Phillips, Karoline Freeman, Julia Geppert, Adeola Agbebiyi, Olalekan A Uthman, Jason Madan, Angus Clarke, Siobhan Quenby, Aileen Clarke

**Affiliations:** 1 Warwick Medical School, The University of Warwick, Coventry, West Midlands, UK; 2 Institute of Cancer & Genetics, Cardiff University School of Medicine, Cardiff, UK

**Keywords:** REPRODUCTIVE MEDICINE

## Abstract

**Objective:**

To measure test accuracy of non-invasive prenatal testing (NIPT) for Down, Edwards and Patau syndromes using cell-free fetal DNA and identify factors affecting accuracy.

**Design:**

Systematic review and meta-analysis of published studies.

**Data sources:**

PubMed, Ovid Medline, Ovid Embase and the Cochrane Library published from 1997 to 9 February 2015, followed by weekly autoalerts until 1 April 2015.

**Eligibility criteria for selecting studies:**

English language journal articles describing case–control studies with ≥15 trisomy cases or cohort studies with ≥50 pregnant women who had been given NIPT and a reference standard.

**Results:**

41, 37 and 30 studies of 2012 publications retrieved were included in the review for Down, Edwards and Patau syndromes. Quality appraisal identified high risk of bias in included studies, funnel plots showed evidence of publication bias. Pooled sensitivity was 99.3% (95% CI 98.9% to 99.6%) for Down, 97.4% (95.8% to 98.4%) for Edwards, and 97.4% (86.1% to 99.6%) for Patau syndrome. The pooled specificity was 99.9% (99.9% to 100%) for all three trisomies. In 100 000 pregnancies in the general obstetric population we would expect 417, 89 and 40 cases of Downs, Edwards and Patau syndromes to be detected by NIPT, with 94, 154 and 42 false positive results. Sensitivity was lower in twin than singleton pregnancies, reduced by 9% for Down, 28% for Edwards and 22% for Patau syndrome. Pooled sensitivity was also lower in the first trimester of pregnancy, in studies in the general obstetric population, and in cohort studies with consecutive enrolment.

**Conclusions:**

NIPT using cell-free fetal DNA has very high sensitivity and specificity for Down syndrome, with slightly lower sensitivity for Edwards and Patau syndrome. However, it is not 100% accurate and should not be used as a final diagnosis for positive cases.

**Trial registration number:**

CRD42014014947.

**Strengths and limitations of this study**

- This is a full systematic review with searches across multiple databases dating back to 1997, and two authors sifting all titles and abstracts.
- Two authors extracted data on prepiloted forms and appraised quality using an adapted QUADAS 2 form.
- The meta-analysis included rigorous methods of data analysis, including bivariate random-effects regression models, but required a zero-cell correction to enable model convergence which may underestimate rather than overestimate accuracy.
- The meta-analysis included a series of subgroup and sensitivity analyses to test for robustness of our pooled diagnostic accuracy estimates.
- The methods are transparent with full protocol published in PROSPERO in advance of the review.

## Introduction

Non-invasive prenatal testing (NIPT) using cell-free fetal DNA (cffDNA) is a method for testing for trisomies in the fetus, using a peripheral sample of the pregnant mother's blood. It is currently marketed across 61 countries in Europe, Asia, Africa and North and South America.^1^ Rapid adoption in the USA has seen increases in first trimester screening using NIPT, and concurrent decreases in the first trimester combined test and invasive testing.^2^ ^3^ People tend to overestimate the usefulness of genetic tests, and misinterpret their meaning.^3^ It is possible that pregnant women will interpret a positive NIPT test as positive diagnosis, and wish to abort a pregnancy on this basis. A clear summary of test accuracy for NIPT is necessary for use by doctors and patients for use in shared and informed decision-making.

Although a previous review of NIPT test accuracy exists,^4^ it does not include two of the largest studies.^5^ ^6^ In addition the authors use a univariate approach which is not appropriate for meta-analysis of tests since it overlooks the fact that sensitivity and specificity are usually negatively correlated across studies due to different thresholds used to define positive and negative test results. It has been shown that ignoring this correlation would be inappropriate.^7^ The weighted sums of the reported specificity are normally used to assess the value of a test, the properties of the resulting statistics depends most importantly on this correlation between the estimates, and it is exactly that is ignored in separate univariate analyses.^8^ ^9^ Most importantly, the previous review does not provide a summary of findings which can be applied to a relevant population and used in clinician–patient shared decision-making.

The UK National Screening Committee commissioned this new review to provide a summary of the accuracy of NIPT for detection of Down, Edwards and Patau syndromes in first trimester pregnancies, to inform their decision on introduction of this test into current fetal abnormality screening in the UK.

## Methods

### Identification and selection of studies

Ethical approval was granted from the University of Warwick Biomedical and Scientific Research Ethics Committee reference REGO-2015-1446. Searches were conducted in PubMed, Ovid Medline, Ovid Embase and the Cochrane Library. The search strategy used a combination of search terms for the NIPT test and trisomies, and was limited to the English language, (see online supplementary file 1). Date limits were 01.01.1997–09.02.2015. Updating autoalerts in Medline and Embase were run until 01.04.2015. Individuals and organisations were contacted for studies not freely available in the public domain. ClinicalTrials.gov, WHO International Clinical Trials Registry Platform (ICTRP) Search Portal and meeting abstracts were also searched for ongoing or recently completed trials.

Two reviewers independently screened titles and abstracts of all records obtained. Discrepancies were resolved by consensus or discussion with a third reviewer. Inclusion criteria were English language journal articles which investigated NIPT using cff DNA derived from maternal blood (serum, plasma, whole blood) in pregnant women in any trimester for the detection of Down (T21), Edwards (T18) or Patau (T13) syndromes in the fetus. The reference standard was genetic verification through amniocentesis, Chorionic Villus Sampling (CVS), cordocentesis, fetal pathological examination after abortion or postnatal phenotypic assessment. We included studies with any outcomes reported on test accuracy, or rates of test failure or indeterminate results. We excluded studies reporting the quantification of fetal cells or DNA or using elevated levels of the whole fetal DNA or epigenetic markers. We also excluded case–control studies with fewer than 15 cases and cohort studies with fewer than 50 pregnant women as well as studies with incomplete 2×2 data or studies which reused samples from other included studies in order to prevent double counting.

Data were extracted by one reviewer and checked by a second reviewer. Disagreements were resolved by consensus or discussion with a third reviewer. Full data extraction forms are available from the authors on request.

### Quality assessment

The quality of diagnostic accuracy studies was assessed using a modified QUADAS-2.^10^ Quality assessment was undertaken by one reviewer and checked by a second reviewer, with disagreements resolved by a third reviewer. Three modifications were made. First, an additional signalling question was added on whether the study avoided taking the sample for the index test in the 7 days after an invasive test, as fetal fraction may be elevated at this time boosting the performance of NIPT. Second, a signalling question was added to determine whether the threshold value was determined using an independent set of samples, and whether adjustment of the predefined threshold was avoided, since the threshold for testing positive is expressed as number of SDs from the mean score for a set of normal samples, rather than as an absolute threshold. Finally, the standard QUADAS-2 signalling question determining whether there was an appropriate interval between index test and reference standard was removed, as timing of an invasive test (apart from in relation to invasive testing) would not affect accuracy. Timing of the NIPT test is important as fetal fraction and therefore accuracy of NIPT increases throughout pregnancy, this was included under applicability of findings rather than as a source of bias. We also assessed the role of the sponsor in addition to QUADAS-2. This included studies that clearly declared involvement of a sponsor in the design or conduct of the study or publication, the majority of authors were employees or shareholders of companies offering NIPT or cytogenetic tests and/or other conflicts of interest (ie, patents, stock or stock options). Please see online supplementary file 2 for full information on the definition for the signalling questions of the QUADAS-2.

### Statistical analysis of test accuracy studies

All eligible studies were included in a meta-analysis of performance of the NIPT test. We extracted data from the primary studies to obtain the four cell values of a diagnostic 2×2 table in order to calculate test accuracy measures. We pooled the sensitivity and specificity estimates using bivariate random-effects regression models, as recommended by the Cochrane Diagnostic Test Accuracy Working Group,^11^ in order to take the potential trade-off between sensitivity and specificity explicitly into consideration and incorporate this negative correlation into the analysis.^7^ We added a 0.5 cell correction to each cell where a zero was encountered. We stratified test accuracy measures according to condition (T21, T18 and T13).

### Meta-analysis, subgroup and sensitivity analyses

We used sensitivity, subgroup and meta-regression analyses to explore potential sources of heterogeneity in test accuracy estimates across studies. The following variables were selected a priori as potential sources of heterogeneity: study design (cohort with consecutive sampling vs others), population risk (general, high-risk, others), population (twins vs others), first trimester (100% vs other), test type (MPSS, DANSR, single nucleotide polymorphism (SNP) technology) and publication year (2007–2013 vs 2014–2015). We conducted a series of sensitivity analyses to check the robustness of the results. We excluded all studies with zero cases of true positive and false negative results. We used Cook's distance to identify particularly influential studies and created a scatter plot of the standardised predicted random effects (standardised level 2 residuals) to check for outliers.^12^ We refitted the model leaving out outliers and very influential studies.

We constructed 3×2 tables to examine the influence of the number of test failures and indeterminate results on the pooled test accuracy estimates.^13^ Test failures occur where the NIPT test has failed to produce any result, and indeterminate results where the test result is in a mid-range which is neither positive nor negative. Test failures can occur for a variety of reasons, and sometimes the cause is unknown. Test failures and indeterminate results are not included in the 2×2 tables reported, and this can lead to overestimates of sensitivity and specificity.^14^ We included all failures of the NIPT test, regardless of whether repeating the test on the same or a new blood sample would have given a result, but we did not include failures which could be rectified by good quality assurance procedures (such as insufficient blood or dropped samples). For the 3×2 tables we considered the following three scenarios, all non-evaluable results: (1) considered to be positive results to reflect use of the NIPT as triage for invasive testing,^14^ (2) considered to be negative results to reflect use of NIPT as an add-on to the combined test^14^ and (3) follow intention to diagnose principle to account for the first two approaches overestimating specificity and sensitivity, respectively.^13^ Intention to diagnose was defined as “including non-evaluable results either in the ‘false negative’ or the ‘false positive’ cell of a 2×2 table (worst case scenario) according to the results of the reference standard”. For the intention to diagnose principle, all non-evaluable positive results were assumed to be false negative and all non-evaluable negative results were assumed to be false positive. Where the reference standard results were not reported for these cases, we assumed that they had the same prevalence of trisomy as those in the rest of the same study.

In the subgroup analyses, we computed pooled accuracy estimates in various strata to determine if accuracy is higher or lower in specific subgroups. Summary sensitivity and specificity estimates for each subgroup were generated, along with their 95% CIs. In the linear meta-regression model, studies are the units of analysis. We used the meta-regression model to generate relative diagnostic ORs.^15^ ^16^ We used Deeks’ funnel plot asymmetry test to test for publication bias, with p value<0.10 indicating significant publication bias.^17^ All analyses were performed using Stata V.13 for Windows including the user written commands metandi, midas, metareg and mvmeta.^12^ ^18–20^

## Results

### Study selection

**Figure 1:**
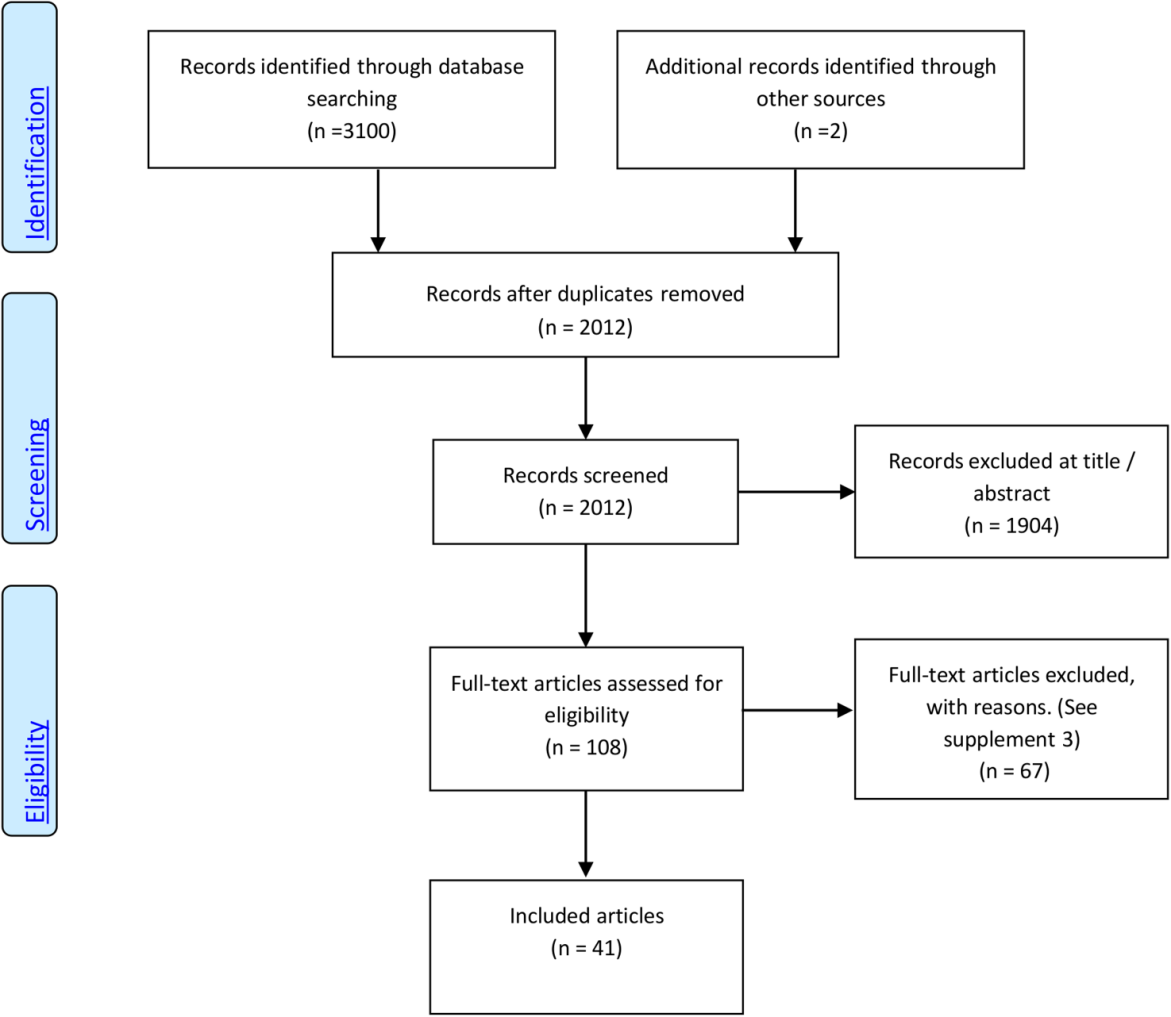
Preferred Reporting Items for Systematic Reviews and Meta-Analyses (PRISMA) flow chart of included articles.

A total of 2012 records were identified after duplicates were removed. One-hundred and eight records remained after evaluation of title and abstract, of which 41 studies were included in the meta-analysis. Figure 1 summarises the study selection process (see online supplementary file 3 for included studies and online supplementary file 4 for reasons of exclusion for 67 full-text articles).

### Characteristics of included studies

#### Study design, populations, reference standards

Forty-one publications, dating from 2007 to 2015, reported NIPT results for between 46 and 112 669 pregnant women for the main autosomal trisomies in relation to fetal karyotype or newborn phenotype and fulfilled our inclusion criteria (see online supplementary file 5). The majority of studies were cohort studies (n=29),^5^ ^6^ ^21–47^ with prospective data collection. There were 11 case–control studies^48–58^ and one of unclear design.^59^ Thirty studies were undertaken in singleton pregnancies only,^6^ ^21^ ^22^ ^29–33^ ^35–37^ ^40–51^ ^53–59^ four studies included singleton and twin pregnancies,^5^ ^28^ ^34^ ^38^ with the remainder undertaken in twin only (n=3).^23^ ^24^ ^39^ In four studies the reporting was unclear.^25^ ^26^ ^28^ ^52^ The majority of studies (n=24) used samples from high-risk pregnant women (positive standard screening, ultrasound abnormalities, advanced maternal age, personal or family history of aneuploidies) undergoing invasive testing.^24^ ^26^ ^28^ ^30^ ^31^ ^33^ ^36–38^ ^41^ ^44^ ^45^ ^47–56^ ^58^ ^59^ Six studies were performed in the general obstetric population.^6^ ^21^ ^29^ ^35^ ^40^ ^43^ Nine studies included pregnant women with mixed risk factors.^5^ ^22^ ^27^ ^32^ ^34^ ^39^ ^42^ ^46^ ^57^ In two the underlying risk was unclear.^23^ ^25^ Seven studies included women in the first trimester only,^6^ ^23^ ^29^ ^30^ ^43^ ^47^ ^48^ while all other studies (n=34) included pregnant women with an unstated, later or broader gestational age window.^5^ ^21^ ^22^ ^24–28^ ^31–42^ ^44–46^ ^49–59^

#### Testing strategies

Three main testing strategies were pursued by the majority of studies (see online supplementary file 6). These were genome-wide massively parallel shotgun sequencing (MPSS, n=24 studies),^5^ ^21^ ^22^ ^24–28^ ^33–36^ ^41^ ^44–47^ ^49–52^ ^54^ ^55^ ^58^ targeted massively parallel sequencing (DANSR, n=9 studies),^6^ ^23^ ^29^ ^31^ ^37^ ^39^ ^43^ ^48^ ^56^ and SNP technology (n=5).^30^ ^32^ ^42^ ^53^ ^57^ Two studies, performed in real clinical settings, offered more than one NIPT approach.^38^ ^40^ Dhallan *et al*^59^ used a DNA-SNP allelic ratio approach.

In 3 of the 41 studies,^21^ ^32^ ^57^ some of the maternal blood samples for NIPT were obtained after invasive testing and for 34 studies we concluded that tests were collected before the invasive testing.^5^ ^6^ ^22–24^ ^26–31^ ^33–52^ ^54^ ^55^ ^58^ In four studies, it was unclear if maternal blood sampling for NIPT was performed before or after an invasive procedure.^25^ ^53^ ^56^ ^59^

Forty studies reported NIPT performance for T21,^5^ ^6^ ^21–49^ ^51–59^ 36 for T18,^5^ ^6^ ^21–36^ ^38–50^ ^53–57^ and 30 studies investigated non-invasive detection of T13.^5^ ^6^ ^21^ ^23^ ^25–28^ ^30^ ^32–36^ ^38–47^ ^49^ ^50^ ^53–55^ ^57^ Twenty-nine studies reported test accuracy for all three main autosomal trisomies.^5^ ^6^ ^21^ ^23^ ^25–28^ ^30^ ^32–36^ ^38–47^ ^49^ ^53–55^ ^57^

### Methodological quality of included studies

**Figure 2:**
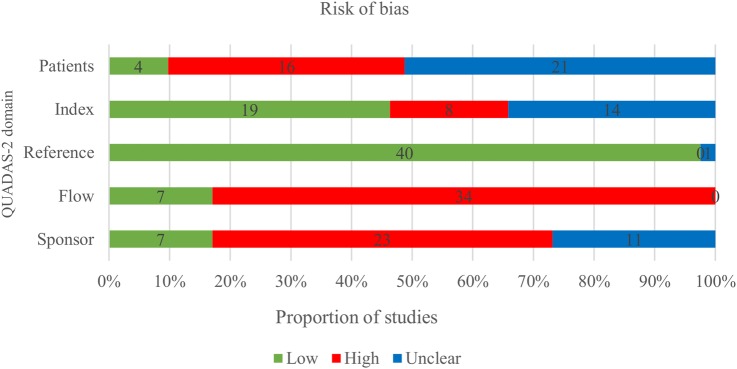
Proportion of studies with low, high or unclear risk of bias using QUADAS 2.

**Figure 3:**
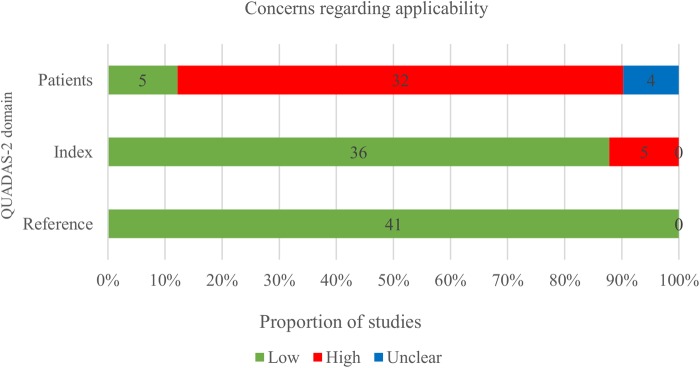
Proportion of studies with low, high and unclear concerns regarding applicability using QUADAS 2.

The methodological quality of the 41 included studies, assessed by QUADAS-2^10^ is summarised in figures 2 and 3 and online supplementary file 7. Risk of bias was high in most studies with 25 of 41 studies considered high risk in two or more domains, and 14 studies in one domain. Two were judged as low or unclear risk of bias in all five domains. Figure 2 shows that study flow (concerned with patient follow-up) and the role of the sponsor were the areas with the greatest risk of bias. Another issue was incomplete or unclear reporting, particularly of the patient selection process and the conduct of the index test, which is reflected in 21 (51.2%) and 14 (34.1%) of 41 publications scoring an unclear risk of bias in these two domains, respectively. The risk of bias regarding the reference standard was considered low in almost all studies with only one study classified as unclear.^23^ Finally, risk of bias regarding the role of sponsor was deemed high in 23 studies. There were significant concerns regarding applicability of the included patient spectrum to cffDNA testing introduction in the first trimester (see figure 3), as 29 of 41 studies had significant parts (>20%) of their populations tested in the second or third trimester when fetal fraction and therefore accuracy of NIPT is higher.

### Meta-analysis

**Figure 4:**
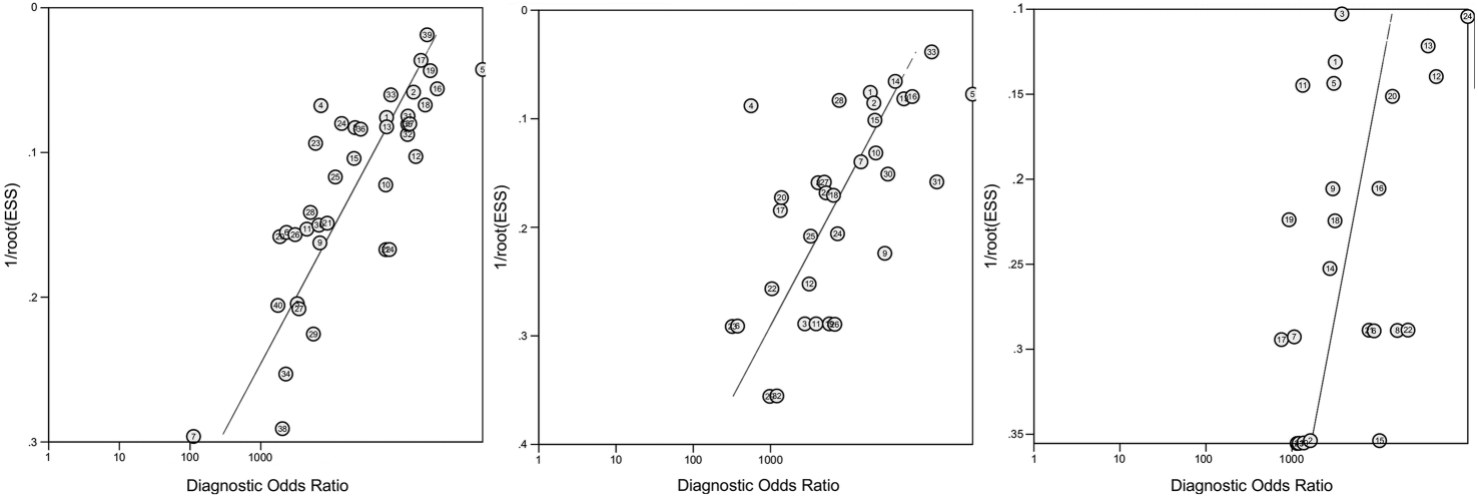
Deeks’ funnel plot for Down (left) Edwards (centre) and Patau (right) syndromes. A vertical pattern would indicate no bias, slope is associated with publication bias.

There was a high likelihood of publication bias, with the slope coefficients on Deeks’ funnel plot asymmetry test significant for Down syndrome (p=0.0001), Edwards syndrome (p=0.0001), and Patau syndrome (p=0.045) (see figure 4).

**Figure 5:**
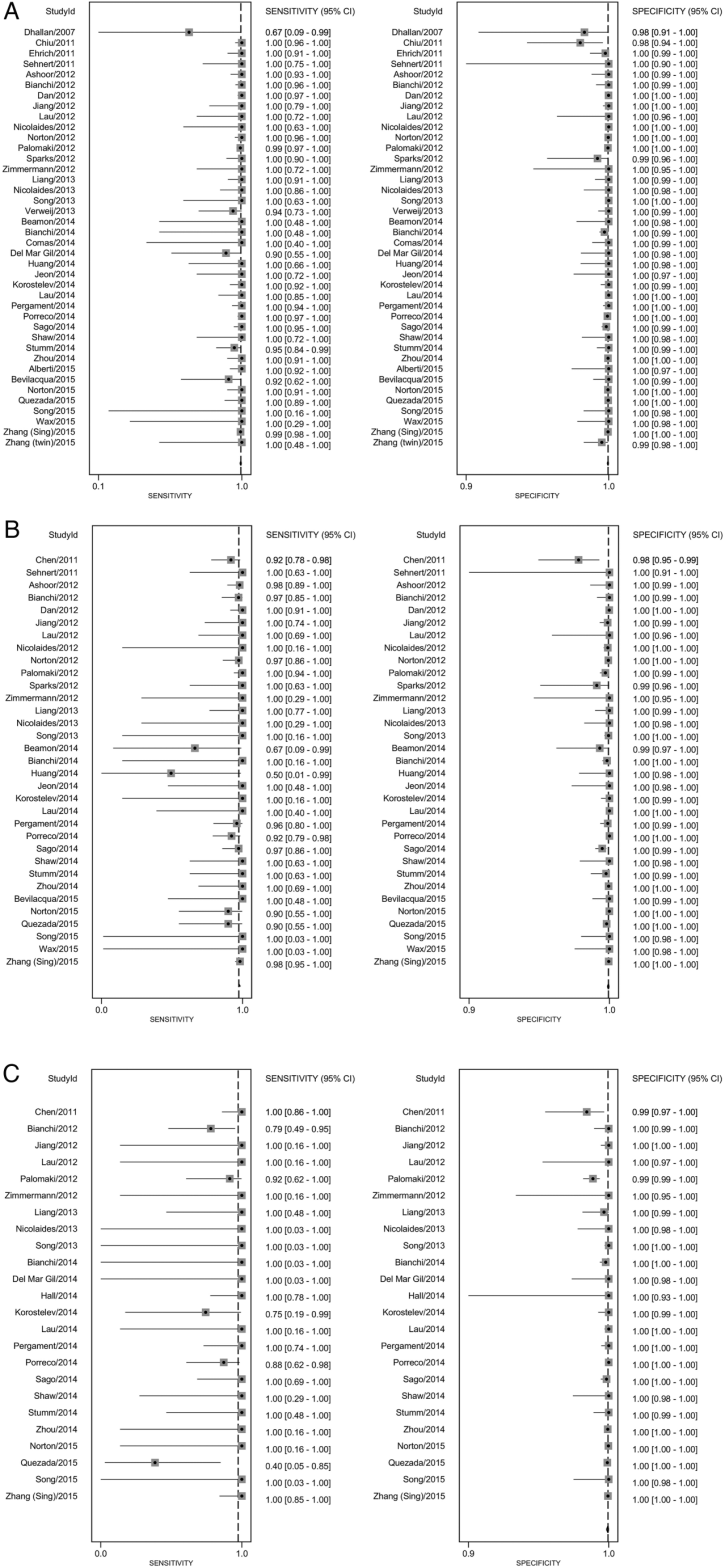
Individual and pooled sensitivity and specificity for non-invasive prenatal testing (NIPT) for the detection of a. Down syndrome b. Edwards syndrome and c. Patau syndrome.

The pooled sensitivity for Down syndrome from bivariate random-effects regression of 40 studies was 99.3% (98.9% to 99.6%) and the pooled specificity was 99.9% (99.9% to 100%). For Edwards syndrome the pooled sensitivity over 33 studies was 97.4% (95.8% to 98.4%) and specificity was 99.9% (99.9% to 100%). For Patau syndrome the pooled sensitivity over 24 studies was 97.4% (86.1% to 99.6%) and specificity was >99.9% (99.9% to 100%). Table 1 shows these pooled sensitivities and specificities applied to populations of pregnant women taking the test. In the subgroup analysis (table 2) sensitivity estimates were lower by 6.1% for Down, 10.6% for Edwards, and 12.3% for Patau syndromes for cohort studies with consecutive sampling in comparison to all other studies which are more likely to be subject to spectrum bias. Test accuracy did not appear to systematically differ between DANSR, MPSS or SNP-based test types or by publication year. Estimates of test sensitivity were higher in high-risk populations, in studies including pregnancies in the second and third trimester, and in singleton pregnancies. In high-risk populations, defined in a variety of ways, pooled sensitivity estimates were 1.4%, 6.5% and 17.8% higher than in the general obstetric population for Down, Edwards and Patau syndromes, respectively. Sensitivity estimates were 1.3%, 1.4% and 11.6% lower in studies recruiting all women in their first trimester of pregnancy in comparison to studies including women later in pregnancy. The outcomes of test accuracy of the included studies are summarised in online supplementary file 8. A forest plot of the sensitivity and specificity from the individual studies with 95% CIs is given in figure 5.

**Table 1:** Summary of findings applied to high risk and general obstetric population

Condition	Summary accuracy	Median prevalence	Outcomes	Positive predictive value	Probability of false negative	Implications
General obstetric population (100 000 pregnancies)						
Down syndrome	Sensitivity=95.9% Specificity=99.9% (6 studies)	0.43%	TP=417 FP=94 TN=99471 FN=18	82%	1 in 5570	With prevalence of 0.4%, 435 of 100 000 pregnancies will be affected by Down syndrome. Of these 417 will be detected and 18 missed by cffDNA. Of the 99 565 who do not have Down syndrome, 94 will receive a false positive result. Therefore 82% of pregnancies which test positive will have Down syndrome
Edwards syndrome	Sensitivity=86.5% Specificity=99.8% (5 studies)	0.10%	TP=89 FP=154 TN=99744 FN=14	37%	1 in 7194	With prevalence of 0.1%, 102 of 100 000 pregnancies will be affected by Edwards syndrome. Of these 89 will be detected and 14 missed by cffDNA. Of the 99 898 who do not have Edwards syndrome, 154 will receive a false positive result. Therefore 37% of pregnancies which test positive will have Edwards syndrome
Patau syndrome	Sensitivity=77.5% Specificity=>99.9% (5 studies)	0.05%	TP=40 FP=42 TN=99906 FN=12	49%	1 in 8506	With prevalence of 0.05%, 52 of 100 000 pregnancies will be affected by Patau syndrome. Of these 40 will be detected and 12 missed by cffDNA. Of the 99 948 who do not have Patau syndrome, 42 will receive a false positive result. Therefore 49% of pregnancies which test positive will have Patau syndrome
High-risk population (10 000 pregnancies)						
Down syndrome	Sensitivity=97% Specificity=99.7% (22 studies)	3.33%	TP=324 FP=31 TN=9636 FN=9	91%	1 in 1054	With prevalence of 3.3%, 333 of 10 000 pregnancies will be affected by Down syndrome. Of these 324 will be detected and 9 missed by cffDNA. Of the 9667 who do not have Down syndrome, 31 will receive a false positive result. Therefore 91% of those who test positive will have Down syndrome
Edwards syndrome	Sensitivity=93% Specificity=99.7% (19 studies)	1.50%	TP=140 FP=26 TN=9824 FN=11	84%	1 in 930	With prevalence of 1.5%, 151 of 10 000 pregnancies will be affected by Edwards syndrome. Of these 140 will be detected and 11 missed by cffDNA. Of the 9850 who do not have Edwards syndrome, 26 will receive a false positive result. Therefore 84% of those who test positive will have Edwards syndrome
Patau syndrome	Sensitivity=95% Specificity=99.9% (11 studies)	0.50%	TP=47 FP=7 TN=9943 FN=3	87%	1 in 4265	With prevalence of 0.5%, 50 of 10 000 pregnancies will be affected by Patau syndrome. Of these 47 will be detected and 3 missed by cffDNA. Of the 9950 who do not have Patau syndrome, 7 will receive a false positive result. Therefore 87% of those who test positive will have Patau syndrome

Median prevalence determined from cohort studies included in meta-analysis for relevant populations. Estimates of sensitivity and specificity are from meta-analysis sub-groups for studies in high risk and general obstetric populations. The systematic review investigated test accuracy of non-invasive prenatal testing using cell-free DNA derived from maternal blood (serum, plasma, whole blood) in pregnant women in any trimester for the detection of Down, Edwards or Patau syndromes in the fetus. The reference standard was genetic verification through amniocentesis, CVS, cordocentesis, fetal pathological examination after abortion and postnatal phenotypic assessment. Findings should be interpreted with caution. Assessment using QUADAS-2 identified high risk of bias in included studies, particularly for selection of women and flow. Deeks’ funnel plots indicated there was high risk of publication bias in included studies. Zero-cell corrections may have reduced accuracy estimates.

cffDNA, cell-free fetal DNA; CVS, Chorionic Villus Sampling; FN, false negative; FP, false positive; TN, true negative; TP, true positive.

**Table 2:** Accuracy estimates from sensitivity and subgroup analyses of the included studies by different study characteristics†

	Down (trisomy 21)			Edwards (trisomy 18)			Patau (trisomy 13)		
Variables	N	SN (95% CI)	SP (95% CI)	n	SN (95% CI)	SP (95% CI)	n	SN (95% CI)	SP (95% CI)
*All studies*	40	0.993 (0.989 to 0.996)	0.999 (0.999 to 1.000)	33	0.974 (0.958 to 0.984)	0.999 (0.999 to 1.000)	24	0.974 (0.861 to 0.996)	1.000 (0.999 to 1.000)
*Sensitivity analyses*									
Excluding outliers‡	37	0.993 (0.989 to 0.996)	1.000 (0.999 to 1.000)	32	0.977 (0.961 to 0.986)	0.999 (0.999 to 1.000)	22	0.977 (0.818 to 0.998)	1.000 (0.999 to 1.000)
Test failures									
Assuming all+ve	40	0.997 (0.990 to 0.999)	0.981 (0.972 to 0.988)	33	0.973 (0.956 to 0.983)	0.983 (0.974 to 0.990)	24	0.979 (0.873 to 0.997)	0.981 (0.966 to 0.989)
Assuming all−ve	40	0.962 (0.948 to 0.973)	1.000 (0.999 to 1.000)	33	0.942 (0.913 to 0.962)	0.999 (0.999 to 1.000)	24	0.885 (0.796 to 0.939)	1.000 (0.999 to 1.000)
Intention to diagnosis	40	0.976 (0.959 to 0.986)	0.981 (0.972 to 0.989)	33	0.958 (0.927 to 0.976)	0.983 (0.973 to 0.990)	24	0.903 (0.811 to 0.953)	0.981 (0.966 to 0.989)
Assuming all+ve	40	0.994 (0.989 to 0.997)	0.999 (0.999 to 1.000)	33	0.974 (0.958 to 0.985)	0.999 (0.999 to 1.000)	24	0.974 (0.863 to 0.996)	1.000 (0.999 to 1.000)
Assuming all−ve	40	0.993 (0.987 to 0.996)	0.999 (0.999 to 1.000)	33	0.970 (0.945 to 0.984)	0.999 (0.999 to 1.000)	24	0.976 (0.855 to 0.996)	1.000 (0.999 to 1.000)
Intention to diagnosis	40	0.993 (0.988 to 0.996)	0.999 (0.999 to 1.000)	33	0.972 (0.950 to 0.985)	0.999 (0.999 to 1.000)	24	0.976 (0.855 to 0.996)	1.000 (0.999 to 1.000)
*Subgroup analyses*									
Study design									
Cohort	5	0.932 (0.853 to 0.971)	0.999 (0.996 to 1.000)	4	0.868 (0.591 to 0.968)	0.998 (0.994 to 0.999)	3	0.851 (0.498 to 0.971)	0.999 (0.995 to 1.000)
Others	35	0.976 (0.963 to 0.985)	0.998 (0.997 to 0.999)	29	0.941 (0.914 to 0.960)	0.998 (0.997 to 0.999)	21	0.970 (0.852 to 0.994)	1.000 (0.999 to 1.000)
Population risk									
General	6	0.959 (0.874 to 0.987)	0.999 (0.998 to 1.000)	4	0.865 (0.627 to 0.961)	0.998 (0.997 to 0.999)	4	0.775 (0.135 to 0.987)§	1.000 (0.999 to 1.000)
High	22	0.973 (0.951 to 0.985)	0.997 (0.994 to 0.998)	19	0.930 (0.892 to 0.955)	0.997 (0.995 to 0.999)	11	0.953 (0.864 to 0.985)	0.999 (0.996 to 1.000)
Others	12	0.974 (0.940 to 0.989)	0.999 (0.998 to 0.999)	10	0.958 (0.907 to 0.982)	0.999 (0.999 to 1.000)	9	0.988 (0.547 to 1.000)	1.000 (0.999 to 1.000)
Population									
Others	36	0.977 (0.965 to 0.985)	0.998 (0.997 to 0.999)	31	0.943 (0.917 to 0.960)	0.998 (0.997 to 0.999)	23	0.974 (0.861 to 0.996)	1.000 (0.999 to 1.000)
Twins	4	0.894 (0.750 to 0.960)	0.996 (0.996 to 0.996)	2	0.737 (0.202 to 0.969)§	0.998 (0.986 to 1.000)	1*		
First trimester									
100%	7	0.960 (0.887 to 0.987)	0.999 (0.998 to 1.000)	5	0.925 (0.814 to 0.972)	0.998 (0.997 to 0.999)	5	0.850 (0.770 to 0.906)§	0.999 (0.998 to 0.999)
Others	33	0.973 (0.958 to 0.983)	0.998 (0.997 to 0.999)	28	0.939 (0.910 to 0.960)	0.998 (0.997 to 0.999)	19	0.966 (0.872 to 0.992)	1.000 (0.999 to 1.000)
Test types									
DANSR	9	0.958 (0.898 to 0.983)	0.999 (0.997 to 1.000)	6	0.948 (0.879 to 0.979)	0.998 (0.996 to 0.999)	3	0.606 (0.216 to 0.895)	1.000 (0.998 to 1.000)
MPSS	25	0.978 (0.963 to 0.987)	0.998 (0.997 to 0.999)	23	0.936 (0.899 to 0.960)	0.998 (0.997 to 0.999)	16	0.959 (0.989 to 0.991)	1.000 (0.999 to 1.000)
SNP technology	4	0.984 (0.937 to 0.996)	0.998 (0.993 to 1.000)	4	0.918 (0.751 to 0.976)	0.998 (0.994 to 1.000)	5	0.870 (0.647 to 0.960)	0.998 (0.992 to 0.999)
Publication year									
2007–2013	18	0.977 (0.958 to 0.988)	0.998 (0.995 to 0.999)	15	0.954 (0.919 to 0.975)	0.998 (0.995 to 0.999)	9	0.933 (0.799 to 0.980)	0.999 (0.993 to 1.000)
2014–2015	22	0.966 (0.939 to 0.981)	0.999 (0.998 to 0.999)	18	0.915 (0.853 to 0.952)	0.996 (0.998 to 0.999)	15	0.984 (0.770 to 0.999)	1.000 (0.999 to 1.000)

*Bivariate model inestimable for only one study in the subgroup.^23^

†Excluded studies with inestimable sensitivity (T21—Hall 2014; T18—Comas 2014, Hall 2014, Zhang (twins) 2015; T13—Sehnert 2011, Beamon 2014, Comas 2014, Bevilacqua 2015, Wax 2015, Zhang (twins) 2015).

‡Excluded outliers (T21—Dhallan 2007, Chiu 2011, Sparks 2012; T18—Chen 2011; T13—Chen 2011, Palomaki 2012).

‡p Value for subgroup differences <0.05 (statistically significant).

SN, sensitivity; SNP, single nucleotide polymorphism; SP, specificity.

### Test failures

The rate of analytic failure (failure of the cffDNA testing) ranged from 0% to 12.7%^57^ and among 5789 pregnancies with resampling, 803 (13.9%) also failed the repeat cffDNA testing. There were five papers in this review that reported indeterminate results (results in a range defined as neither positive nor negative) for trisomies 21, 18 and 13.^21^ ^38^ ^49^ ^55^ ^60^ ranging from 0% (0/2042) to 11.1% (5/45). In the study with no indeterminate results they used eight-plex testing, and where the initial score was indeterminate they repeated using one-plex which corrected any indeterminate results. There is some evidence that the rate of test failure is higher when gestational age is lower, and in trisomic pregnancies. Pergament *et al*^32^ found that failure rate at <9 weeks was 26/95 (27.4%), between 9.0 and 9.9 weeks was 6/50 (12.0%), and more than 10 weeks was 53/900 (5.9%). The same study found aneuploidy incidence was increased (20/86 (23.3%)) in samples that did not return a result when compared with the aneuploidy incidence in samples with a cffDNA testing result (105/966 (10.9%), p=0.004). Norton *et al*^6^ did not find an association between test failure and gestational age in 18 510 women between 10 and 14 weeks gestation, but found that the prevalence of aneuploidy in the group with test failure (1 in 38 (2.7%)) was higher than the prevalence of 1 in 236 (0.4%) in the overall cohort (p<0.001).

Including test failures in an intention to diagnose analysis in the meta-analysis decreased sensitivity estimates by 1.7% for Down, 1.6% for Edwards and 7.1% for Patau syndrome, and decreased specificity estimates by nearly 2% for all three trisomies. Excluding test failures from the calculations of test accuracy may have caused overestimation of accuracy. Similarly in the subgroup analysis sensitivity estimates were lower by 6.1% for Down, 10.6% for Edwards, and 12.3% for Patau syndromes for cohort studies with consecutive sampling in comparison to all other studies. Test accuracy did not appear to differ systematically between DANSR, MPSS or SNP technology, or by publication year. Estimates of test sensitivity were higher in high-risk populations, in studies including pregnancies in the second and third trimester, and in singleton pregnancies. In high-risk populations, defined in a variety of ways, pooled sensitivity estimates were 1.4%, 6.5% and 17.8% higher than in the general obstetric population for Down, Edwards and Patau syndromes, respectively. Sensitivity estimates were 1.3%, 1.4% and 11.6% lower in studies recruiting all women in their first trimester of pregnancy in comparison to studies including women later in pregnancy. Twin pregnancies had 8.3% lower sensitivity estimates than singletons for Down syndrome. This difference was 20.6% for Edwards syndrome, but there was only one study for Patau syndrome so we were unable to provide a pooled estimate for twins. Sensitivity and subgroup analyses are reported in table 2.

## Discussion

In a systematic review of 2012 articles, we identified 41 articles on the test accuracy of NIPT. Quality appraisal using QUADAS-2 indicated high risk of bias, in particular due to unclear or unsystematic inclusions and exclusions of participants at study entry level as well as at the level of analysis. Applicability of findings was of concern as there is still very limited data on the screening population available. Pooled sensitivity from the meta-analysis was 99.3% for T21, 97.4% for T18 and 97.4% for T13, with pooled specificity 99.9% (99.9% to 100%) for all three trisomies. We estimated test accuracy in a high-risk population of 10 000 pregnancies where 3.3% of fetuses have Down syndrome, 1.5% have Edwards syndrome and 0.5% have Patau syndrome. There would be 324 cases of Down syndrome detected, with 9 missed and 31 false positive results, 140 cases of Edwards syndrome detected with 11 missed and 26 false positive results, and 47 cases of Edwards syndrome detected, with 3 missed and 7 false positive results (table 1). In the general obstetric population where prevalence of trisomy is lower, there would be a lower positive predictive value. In 100 000 pregnancies in the general obstetric population we would expect 417, 89 and 40 cases of Downs, Edwards and Patau syndromes to be detected by NIPT, with 94, 154 and 42 false positive results. Therefore it is vital to follow a positive NIPT test with an invasive diagnostic test (amniocentesis or CVS) to confirm the presence of trisomy, if the woman is considering termination of pregnancy on the basis of trisomy.

The strengths of this systematic review included a comprehensive search of the literature, with quality appraisal of all included studies, with two authors sifting studies for inclusion, extracting data and appraising quality. The meta-analysis included rigorous methods of data analysis, including bivariate random-effects regression models and HSROC curve analysis. We also conducted a series of subgroup analyses and sensitivity analyses to test for robustness of our pooled diagnostic accuracy estimates. Homogeneous subgroup and sensitivity analysis summary accuracy estimates were generally similar to the overall estimates. We added predefined covariates to the model using meta-regression analyses to explain heterogeneity but considerable statistical heterogeneity remained. For some of the subgroup analyses, the relatively small number of studies available limited the generalisability of such pooled accuracy estimates. Finally we applied zero cell continuity correction of 0.5 to each cell of a study where a zero is encountered which tends to underestimate rather than overestimate test accuracy.

The findings of our review are in line with the results from previous reviews stating that NIPT has high performance in terms of sensitivity and specificity,^61^ ^62^ that specificity is slightly higher than sensitivity,^61^ that the test performance is greater for T21 than for T18 and T13,^4^ and that NIPT is less successful in twin pregnancies than in singleton pregnancies.^4^ However, we found evidence of significant publication bias, converted results into a format interpretable by clinicians, and concluded that the test is not diagnostic. There are two key differences between our review and the previous publications. First, we included more studies, several of which have been published since the most recent review^4^ (including two of the largest studies with test accuracy for 128 510 women).^5^ ^6^ Second, the two previous reviews conducted separate pooling of the diagnostic test accuracy measures using a univariate approach using standard methods for proportion^4^ ^62^ which is not recommended for reviews of test accuracy. Berkey *et al*^63^ show that a bivariate meta-regression is more efficient than separate univariate meta-regressions for assessing study-level covariates, due to the inclusion of correlation. We used Deeks’ funnel plots and found evidence of publication bias, whereas the previous review used an Egger's bias applied to sensitivity and specificity separately and found no evidence of bias, although their method may not be appropriate for studies of test accuracy.^17^ Studies with a larger effective sample size tended to report higher diagnostic ORs. This may be due to publication bias in large laboratory cohort or case–control studies with a lack of systematic or consecutive sampling, or the fact that studies in the general obstetric population tend to have lower test accuracy and fewer cases. It may be partly due to our methods in that the zero-cell correction may disadvantage small studies, or simply that the test is performed to a higher standard in larger studies, perhaps due to more advanced protocols used in later large scale studies.

The implications for policymakers and clinicians are that NIPT using cffDNA has very high sensitivity and specificity, and can contribute to screening programmes for Down, Edwards and Patau syndromes. It is clear that test accuracy is very good but not perfect. This is particularly true when considering populations in terms of risk and gestational age. Our subgroup analyses showed that test performance is better in high-risk populations as well as in studies including pregnancies in the second and third trimester. Consideration of NIPT as a screening test for the general obstetric population primarily tested in the first trimester of pregnancy has to take into account the lower sensitivity of NIPT in this population. There is also some indication that higher maternal weight, and conception by in vitro fertilisation (IVF) are potential predictors of NIPT test failure^39^ suggesting that NIPT may not work equally well in all subpopulations. We consider that for this reason cffDNA should not be regarded as a diagnostic test and that confirmation of a positive NIPT result by amniocentesis or CVS is necessary to make a diagnosis of trisomy. This is essential if parents are considering termination of pregnancy on the basis of trisomy, because in the general obstetric population as many as 20% of positive NIPT results for Down syndrome may be false positive. This proportion will be higher for Edwards and Patau syndromes. Because the source of cffDNA is the placenta, confined placental mosaicism may explain a proportion of discordant NIPT results.^64^ Furthermore, early fetal demise of an affected fetus^53^ ^64^ and unknown chromosomal abnormality in the mother^5^ ^64^ can lead to false positive results. Finally, in some cases discordance between NIPT and fetal karyotype results might be due to lab error.^64^ The role of low fetal fraction as contributor to false positive or false negative results is unclear: Zhang *et al*^5^ reported no major influence, whereas Quezada *et al*^43^ found lower fetal fractions in discordant than in those with concordant results.

Communicating to clinicians and patients that this genetic test is not perfect will be key for safe implementation, and pretest and post-test information provision and counselling for positive and negative NIPT results should be given careful consideration. The NIPT test may be particularly attractive to parents who are not considering termination of pregnancy, but who would like to know in advance if their pregnancy is affected by a trisomy, since NIPT gives broadly accurate results, without the slightly increased risk of miscarriage associated with invasive procedures such as amniocentesis and CVS. The final consideration for implementation is the range of test failure rates from <1% to >12%, with some evidence that presence of trisomy may be a predictor of test failure. Quality assurance to minimise test failures would minimise delays due to repeated testing, which may be a priority for pregnant women. However, if the test failure is due to insufficient fetal fraction a retest is also likely to fail.

This test is used worldwide, mostly provided directly by private providers rather than national health systems. Further research into how the test is being interpreted and understood by clinicians and pregnant women will be key to understanding the balance of benefits and harms from the provision of the test. In particular, how this understanding leads to decisions about whether to continue the pregnancy, and whether this may be influenced by how the test is presented to parents both by companies, and by clinicians. Finally if it is implemented into national screening programmes, keeping accurate records of outcomes and test failures would enable the test performance to be evaluated in practice. This may differ from the test accuracy in the included studies in this paper, due to the high risk of bias in included studies of cffDNA, and the unexplained heterogeneity illustrating the uncertainties in transferring results from research studies into everyday practice.
